# How did the domestication of Fertile Crescent grain crops increase their yields?

**DOI:** 10.1111/1365-2435.12760

**Published:** 2016-10-03

**Authors:** Catherine Preece, Alexandra Livarda, Pascal‐Antoine Christin, Michael Wallace, Gemma Martin, Michael Charles, Glynis Jones, Mark Rees, Colin P. Osborne

**Affiliations:** ^1^ Department of Animal and Plant SciencesUniversity of SheffieldSheffieldS10 2TNUK; ^2^ CREAFCampus de Bellaterra (UAB)Edifici C08193Cerdanyola del VallèsSpain; ^3^ Department of ArchaeologyUniversity of NottinghamNottinghamNG7 2RDUK; ^4^ Department of ArchaeologyUniversity of SheffieldSheffieldS1 4ETUK; ^5^ Institute of ArchaeologyUniversity of OxfordOxfordOX1 2PGUK

**Keywords:** cereal, crop progenitors, domestication, Fertile Crescent, legume, origins of agriculture, size, yield

## Abstract

1. The origins of agriculture, 10 000 years ago, led to profound changes in the biology of plants exploited as grain crops, through the process of domestication. This special case of evolution under cultivation led to domesticated cereals and pulses requiring humans for their dispersal, but the accompanying mechanisms causing higher productivity in these plants remain unknown. The classical view of crop domestication is narrow, focusing on reproductive and seed traits including the dispersal, dormancy and size of seeds, without considering whole‐plant characteristics. However, the effects of initial domestication events can be inferred from consistent differences between traditional landraces and their wild progenitors.
2. We studied how domestication increased the yields of Fertile Crescent cereals and pulses using a greenhouse experiment to compare landraces with wild progenitors. We grew eight crops: barley, einkorn and emmer wheat, oat, rye, chickpea, lentil and pea. In each case, comparison of multiple landraces with their wild progenitors enabled us to quantify the effects of domestication rather than subsequent crop diversification. To reveal the mechanisms underpinning domestication‐linked yield increases, we measured traits beyond those classically associated with domestication, including the rate and duration of growth, reproductive allocation, plant size and also seed mass and number.
3. Cereal and pulse crops had on average 50% higher yields than their wild progenitors, resulting from a 40% greater final plant size, 90% greater individual seed mass and 38% less chaff or pod material, although this varied between species. Cereal crops also had a higher seed number per spike compared with their wild ancestors. However, there were no differences in growth rate, total seed number, proportion of reproductive biomass or the duration of growth.
4. The domestication of Fertile Crescent crops resulted in larger seed size leading to a larger plant size, and also a reduction in chaff, with no decrease in seed number per individual, which proved a powerful package of traits for increasing yield. We propose that the important steps in the domestication process should be reconsidered, and the domestication syndrome broadened to include a wider range of traits.

## Introduction

The origins of agriculture transformed human societies and drove some of the most important cultural changes in human history (Lev‐Yadun, Gopher & Abbo 2000). Understanding why agriculture began is thus one of the most fundamental questions in archaeology, but the mechanisms behind it remain a subject of debate (Abbo, Lev‐Yadun & Gopher 2010a; Fuller, Willcox & Allaby 2011; Price & Bar‐Yosef 2011). Insight may be gained into this process through greater understanding of the changes that plants underwent during crop domestication. The Fertile Crescent in western Asia was one of the major centres of plant domestication, and a number of cereals, including wheat and barley, and several pulses (grain legumes), originated there approximately 10 000 years ago.

A defining characteristic of domesticated seed crops is a loss of natural seed dispersal, whereby plants become totally dependent on people (Fuller 2007). This occurs through indehiscence (inability to shed seed at maturity) of either the spike (in cereals) or the pod (in legumes), which ensures that ripe seeds remain on the plant rather than falling to the ground. Genetic mutations for indehiscent spikes increased in frequency in a small number of wild grass species, firstly in the progenitors (the closest wild relatives) of the primary domesticates barley, einkorn wheat and emmer wheat (approximately 10 000 bp). This change also occurred in rye (some reports of 9000 years bp, but more commonly from 4000 years bp) and oats (some reports of 7000 years bp, but more commonly from 4000 years bp), which are thought to be (especially in the case of oat) secondary domesticates arising later as weeds of cultivation (Zohary, Hopf & Weiss 2012). This distinction between primary and secondary is made because it is possible that some aspects of the domestication process may have been different when occurring for the first time, compared with those occurring later. The pods of legume species are rarely found in archaeological remains, but lentil, pea, chickpea and bitter vetch are thought to have been domesticated around 10 000 bp, and Celtic bean later (possibly 7000 years bp, but more certainly from 4000 years bp) (Zohary, Hopf & Weiss 2012).

In addition to indehiscence, there are also a number of reproductive and regenerative traits typically associated with domesticated plant species and referred to as the ‘domestication syndrome’ (Hammer 1984). There is a substantial literature on the domestication syndrome, particularly for cereals, which have greater seed size, lower seed dormancy, synchronous tillering and maturation, more compact growth, and a reduction in dispersal traits in comparison with their wild progenitors (Harlan, de Wet & Price 1973; Hammer 1984; Fuller 2007; Brown *et al*. 2009). The fact that similar domestication traits are present in unrelated species indicates that these traits arose multiple times independently (Paterson *et al*. 1995; Meyer, DuVal & Jensen 2012).

Traits relating to other aspects of plant growth and yield are not often discussed as part of the domestication syndrome, but may also be important during the domestication process. Indeed, phenotypic integration may mean that selection for one trait results in selection for other traits as well (Murren 2002; Milla *et al*. 2015), a concept that was originally introduced in the context of domestication by Darwin (1859), and which has received experimental support from animal studies (Trut, Oskina & Kharlamova 2009). Identification of these additional traits would allow us to expand the domestication syndrome and reconsider the key steps in the domestication process. In particular, high yield in comparison with wild species is generally considered a significant characteristic of seed crops (Harlan, de Wet & Price 1973; Harlan 1992). We hypothesize that increased yield is a product of other correlated traits such as plant size, arising from either deliberate artificial breeding or unconscious selection by farmers.

Yield can be decomposed in two ways. First, in terms of seed size, the rate and duration of growth, and the allocation of biomass to seeds verses vegetative tissues. The duration of growth is positively correlated with total biomass and yield, for example in durum wheat (Gebeyehou, Knott & Baker 1982), spring bread wheat (Sharma 1992), pearl millet (Craufurd & Bidinger 1988) and oilseed rape (Sidlauskas & Bernotas 2003). Any increase in growth rate should also increase yield, provided that this does not negatively impact other traits. If reproductive allocation increases with domestication, this will also positively influence yield, although evidence that this occurs is mixed. Some studies have found that greater reproductive allocation causes higher yields in crops compared with wild species (Gifford & Evans 1981), but this effect depends on other factors such as plant density and size (Qin *et al*. 2013). Additionally, a decrease in the proportion of chaff leads to higher yield because more of the reproductive biomass is converted into edible seed (Harlan 1992). These components of yield related to size, growth and allocation are not expected to show consistent patterns of covariance.

The second way of decomposing seed yield is to consider the mass and number of individual seeds and to look at how they are packaged into infructescences (i.e. cereal spikes or legume pods) on the plant. Total seed yield increases with greater individual seed mass or a higher number of seeds per plant, and previous research suggests that both traits are important determinants of yield in elite crop varieties (Schwanitz 1966; Evans 1993). However, in this case, we might expect a trade‐off between seed mass and seed number, as commonly observed across a number of different plant species (Sadras 2007; Gambin & Borras 2010). Trade‐offs may be defined as a compromise between how a finite amount of resources is invested in different functions; however, if plant size varies, then resource level also varies. Therefore, this trade‐off will only occur if plants are roughly equal in size, or if biomass‐corrected ratios are used, otherwise no or even positive relationships can occur (Rees & Venable 2007).

The yield advantage of Fertile Crescent crop progenitors over other wild species is usually attributed directly to the fact that these crop progenitors have larger seeds (e.g. Blumler 1998). However, whether a yield advantage was already present in landraces, before agronomic improvement, has not been tested. Although landraces have been evolving during the thousands of generations since domestication, they are certainly our best living proxy for earliest domesticates as they are largely the product of their natural environment and traditional agricultural methods (FAO, 2013), rather than of modern selective breeding techniques (Hedden 2003). Extrapolation from modern crops is unwise, as the process of domestication may be very different from the later process of agronomic improvement, which has led to the development of much higher yielding varieties compared with landraces (Abbo, Lev‐Yadun & Gopher 2010b). It has recently been proposed that traits showing clear and consistent differences between domesticated landraces and their wild progenitors indicate changes from the original domestication episode, rather than subsequent post‐domestication evolution (Abbo *et al*. 2014). Therefore, if we see consistent effects across all of the landraces in comparison with wild progenitors, we can infer that these arise from domestication.

Recent work on wild Fertile Crescent grasses and legumes has produced conflicting evidence over whether crop progenitors produce higher yields than smaller seeded wild species from the same region. Whilst one study looking at nine Fertile Crescent grasses found that crop progenitors had, on average, higher potential grain yields (Cunniff *et al*. 2014), a later study including a larger number of species (24 grasses and 19 legumes) found no such yield advantage (Preece *et al*. 2015). This unexpected result arose from trade‐offs between seed number and mass, and in cereals, also between spike number and mass, that is crop progenitors had larger seeds and spikes but fewer of them (Preece *et al*. 2015). If these trade‐offs are also present within domesticated cereal and pulse species, crops may not necessarily be higher yielding than their wild progenitors and, if they do have higher yields, these may arise from changes in growth or allocation, rather than a direct effect of having larger seeds.

There is also reason to believe that seed size could impact crop yield indirectly, through an effect on overall plant biomass, as previous studies have found positive correlations between these traits. For example, field trials with wheat showed that larger seeds produced plants of greater biomass and height, with higher yields (Donald 1981; Chastain, Ward & Wysocki 1995). It is well‐established for a wide range of species that juvenile plant size is predominantly controlled by seed size, such as between accessions of wild and cultivated barley species (Chapin, Groves & Evans 1989), between 32 species from arid central Australia (Jurado & Westoby 1992) and between 58 British semi‐woody species (Cornelissen 1999). However, correlations between seed size and plant size at maturity are typically weaker (Rees & Venable 2007), indicating that the importance of plant final size in the domestication of crops requires further investigation.

In this paper, we test the hypothesis that domestication has increased seed yield in the landraces of Fertile Crescent cereal and pulse crops. We investigate which yield components are responsible for these increases, hypothesizing that any change in yield might be mediated via trade‐offs or positive correlations among its components. We carried out a comparative experiment in a common greenhouse environment, where cereal and pulse crops and their progenitors were grown individually to maturity. For each crop species, we used multiple landrace accessions, which represent some of the diversity in the least improved extant forms of domesticated species. These are therefore much more closely related to the earliest crops than modern cultivars (McCouch 2004), which is important as it allows inference about the early domesticated states.

## Materials and methods

### Plant Material

For our experiments, we used the landraces of three cereal and three pulse crops known with certainty to have been domesticated at early sites in the Fertile Crescent: barley (*Hordeum vulgare* subsp. *vulgare*), einkorn wheat (*Triticum monococcum* subsp. *monococcum*), emmer wheat (*Triticum turgidum* subsp. *dicoccon*), chickpea (*Cicer arietinum*), pea (*Pisum sativum* subsp. *sativum*) and lentil (*Lens culinaris* subsp. *culinaris*) (Zohary, Hopf & Weiss 2012). In addition, we included oats (*Avena sativa*) and rye (*Secale cereale*), which were also domesticated, probably at a later date and not necessarily in the Fertile Crescent. This may or may not have affected the traits that changed in cereal crops during the domestication process, and there may be ecological reasons why oats and rye did not become domesticated at the same time as wheat and barley. Differences between these two groups of cereal crop progenitors are therefore also interesting. We also used the wild progenitors for each crop, resulting in a total of 10 grasses and seven legumes (Table 1, with more details in Tables S1 and S2, Supporting Information), with two putative pea progenitors included (*P. sativum* subsp. *elatius* and *P. sativum* subsp. *elatius* var. *pumilio*), due to debate in the literature over the closest wild relative (Smykal *et al*. 2011; Zohary, Hopf & Weiss 2012). Seeds for each of the study species were acquired from a number of different seed banks: The National Plant Germplasm System (United States Department of Agriculture), the John Innes Centre Germplasm Resources Unit (UK) and IPK Gatersleben Genebank (Germany). The accessions originated predominantly from western Asia.

**Table 1:** Summary of the 17 species used in this study and their domestication status (crop or progenitor), noting whether each crop and its progenitor are primary (1°) or secondary (2°) domesticates. Primary domesticate denotes one of the first species to be domesticated (*c. *10 000 years ago), whereas secondary domesticate refers to a species thought to be domesticated much later, possibly as weeds of cultivation

Species	Domestication status
*Avena sativa*	Crop (2° domesticate)
*Avena sterilis*	Progenitor (2° domesticate)
*Cicer arietinum*	Crop (1° domesticate)
*Cicer reticulatum*	Progenitor (1° domesticate)
*Hordeum vugare* subsp. *spontaneum*	Progenitor (1° domesticate)
*Hordeum vulgare* subsp. *vulgare*	Crop (1° domesticate)
*Lens culinaris* subsp. *culinaris*	Crop (1° domesticate)
*L. culinaris* subsp. *orientalis*	Progenitor (1° domesticate)
*Pisum sativum* subsp. *elatius*	Progenitor (1° domesticate)
*P. sativum* subsp. *elatius* var. *pumilio*	Progenitor (1° domesticate)
*P. sativum* subsp. *sativum*	Crop (1° domesticate)
*Secale cereale* subsp. *cereale*	Crop (2° domesticate)
*Secale vavilovii*	Progenitor (2° domesticate)
*Triticum monococcum* subsp. *aegilopoides*	Progenitor (1° domesticate)
*Triticum monococcum* subsp. *monococcum*	Crop (1° domesticate)
*Triticum turgidum* subsp. *dicoccoides*	Progenitor (1° domesticate)
*Triticum turgidum* subsp. *dicoccon*	Crop (1° domesticate)

### Growth Conditions

Two greenhouse experiments were conducted in summer 2011 and summer 2013 (described in Preece *et al*. 2015) in order to measure the components of yield, and these are referred to as Yield Experiment 1 and Yield Experiment 2. In 2011, a functional growth analysis was also carried out in a separate study to further understand differences in growth rates between crops and their progenitors and is hereafter called the growth analysis experiment.

In all cases, cereal seeds had outer glumes removed where necessary. For pulses, scarification with sandpaper was used to break seed dormancy. Seeds were germinated on a 1 : 1 mixture of John Innes no. 2 compost (LBS Garden Warehouse, Lancashire, UK) and Chelford 52 washed sand (Sibelco UK Ltd, Cheshire, UK). The growth medium was saturated with water, and seeds were planted in rows to enable identification of individuals. Seeds were germinated in a controlled‐environment growth cabinet (Conviron BDW 40; Conviron, Winnipeg, MB, Canada). Temperature range was 20 °C/10 °C (day/night), with an 8‐h photoperiod and photosynthetic photon flux density (PPFD) of 300 μmol m^−2^ s^−1^, conditions which approximate the growing season for winter annuals in the Fertile Crescent. Seedlings used in Yield Experiments 1 and 2 were transferred to another growth cabinet when they reached the two‐leaf stage, where they were vernalized for 6–8 weeks (the variation was due to small differences between species and between years) to enable subsequent flowering. In this cabinet, the temperature was 4 °C and PPFD 300 μmol m^−2^ s^−1^ with an 8‐h photoperiod. After vernalization, plants were moved to a greenhouse (Arthur Willis Environment Centre, University of Sheffield, Sheffield, UK), and individuals planted into 11‐L square pots (21 × 21 × 25 cm), whilst the temperature was maintained at 24 °C/15 °C (day/night).

In the growth analysis experiment, a vernalization period was not needed, as the experiment was concerned with the initial phase of rapid vegetative growth and not with seed production. Therefore, 3 days after germination, twelve seedlings were randomly selected from those which had successfully germinated. Seedlings were transferred to 1‐L pots containing washed sand and returned to the controlled‐environment room with the following conditions: 20 °C/10 °C (day/night) with a 16‐h photoperiod, maximum PPFD of 756 μmol m^−2^ s^−1^.

### Experimental Design

Yield Experiment 1 used a randomized block design with 20 blocks divided between three greenhouse rooms. Watering occurred three times per week, and plants received Long Ashton nutrient solution (50% concentration) twice during the experiment (Hewitt 1966; tables 40, 41). The two *Secale* species (*Secale vavilovii* and *S. cereale*) do not self‐pollinate, and manual cross‐pollination was therefore carried out using a paintbrush. A subset of cereal spikes (at least five per plant) was covered with translucent, cellophane crossing bags (Focus Packaging and Design Ltd, Scunthorpe, UK), to prevent seed dispersal in the wild species (through natural shattering of the brittle rachis). These bags are specially designed for use with cereals and no differences were observed between bagged and un‐bagged spikes. Crossing bags were not used for wild legume species – instead, seeds were harvested as soon as they were ripe (prior to shattering).

In Yield Experiment 2, the experimental set‐up was the same as the first experiment, except that there were ten blocks in total, divided between two greenhouse rooms. In both years, each block contained one individual of each species where possible, so in total there were up to 30 replicates per species. The *Avena* and *Secale* species were not used in Yield Experiment 2 so the maximum total number of replicates was 20. Replicates were divided approximately equally between accessions in both years.

In the growth analysis experiment, two identical experiments were established, each with the same experimental set‐up, but with different accessions used (see Tables S1 and S2). There were 12 plants per accession, divided between six randomized blocks and pots were top‐watered with full strength Long Ashton solution (Hewitt 1966; tables 40, 41) every 2 days and bottom‐watered with distilled water on alternate days.

### Trait Measurements

In Yield Experiment 1, the duration of the growing period (from germination to flowering) was measured. Final above‐ground biomass was harvested at the end of the experiment, when spikes and pods had reached maturity. Plants were divided into vegetative and reproductive tissues, then oven dried at 40 °C for 3 days and weighed. Allocation to reproductive biomass was calculated as the proportion of the total biomass allocated to reproduction, including culm and chaff. Reproductive biomass was further divided into grain and chaff. The mean individual seed mass was measured before sowing and then again from the harvested seed, calculated from a subset of the infructescences for each plant. Total seed number per plant was also measured, and thus, total seed yield was calculated as the product of mean individual seed mass and the total number of seeds per plant. In cereals but not pulses, the number of seeds per infructescence, the number of infructescences per plant and the total mass of seeds per infructescence were also measured. Maximum plant height (when fully extended) of mature plants was also recorded for cereals and pulses. In Yield Experiment 2 individual seed mass (of sown seed), total seed yield and total above‐ground biomass were measured following the same methods as before. Therefore, for these measurements, data are combined from the 2 years.

For the growth analysis experiment, six harvests were carried out within a 3‐week period, starting on day 8 or 9 after germination and proceeding every 3–4 days, finishing on day 27 or 28. At each harvest, two plants of each species were removed from the pots, washed clean and divided into roots, leaves and stems (in grasses defined as leaf sheath plus culm). Plants were dried to a constant weight for 3 days at 45 °C, and then, dry weight was determined. All species were determined to be in the exponential growth phase between the first and final harvests.

### Components of Yield

Using the results from the Yield Experiments 1 and 2, we investigated yield (total mass of seed) by decomposing it into its separate components. Total seed yield (*Y*) can be calculated in two ways, first:

(eqn 1) $$Y=M_{s}\times \exp\left(\tilde{\lambda }d\right)\times A_{r}\times \left(1-c\right)$$

where *M*_s_ is mean individual seed mass at sowing (g), $\tilde{\lambda }$ is relative growth rate (RGR, g g^−1^ day^−1^) averaged over the growth period, *d* (days), *A*_r_ is allocation to reproductive biomass (dimensionless fraction) and *c* is the proportion of chaff or pods in reproductive biomass (dimensionless fraction). These five components of yield, *M*_s_, *d*, $\tilde{\lambda }$, *A*_r_ and *c*, may covary in some cases. We note that the ‘harvest index’ is a measurement often used in agricultural contexts and can be calculated as the mass of grain (*Y*) as a proportion of above‐ground biomass.

The second way of decomposing seed yield is:

(eqn 2) $$Y=M_{s}\times N_{s}\times N_{i}$$

where *M*_s_ is again mean individual seed mass, *N*_s_ is the number of seeds per infructescence (where infructescence refers to the fruiting part of the plant, either spike, panicle or pod), and *N*_i_ is the number of infructescences per plant. For this equation, *M*_s_ refers to individual seed mass measured at the time at final harvest. In general, sown and harvested individual seed mass are highly correlated with a *~*1:1 relationship (Fig. S1). From eqn 2, total seed yield would increase with greater individual seed mass (*M*_s_) or a higher number of seeds per plant (the product of seed number per infructescence and the number of infructescences per plant, *N*_s_ × *N*_i_).

### Calculation of Growth Rate

We calculated RGR using two approaches. First, average RGR, $\tilde{\lambda }$, was calculated using the final harvest data from Yield Experiments 1 and 2,

(eqn 3) $$\tilde{\lambda }=\frac{\ln\left(\frac{M_{d}}{M_{s}}\right)}{d}$$

where *M*_s_ is the individual seed mass at sowing and *M*_d_ is the final plant mass at the end of the growing period. Note this method of estimating average RGR is valid even when growth is not exponential, and if we substitute this into eqn 1, we find *Y* = *M*_d_ *A*_r_(1 − *c*) as expected. Seed mass is used here as a measure of initial mass, as we are particularly interested in the efficiency with which seed mass is converted into final plant mass. This method therefore differs from the usual way of calculating RGR, although previous work has shown a strong correlation between seedling mass and seed mass in these species (Cunniff *et al*. 2014). The advantage of this method is that it averages across the entire growth period and does not just look at seedlings. Nonetheless, care should be taken interpreting RGR data calculated in this way when there is large variation in initial seed size. RGR calculated in this way does not account for differences in plant size, which makes comparisons between taxa ambiguous as differences may arise from size‐related effects rather than intrinsic differences in maximum RGR (Rees *et al*. 2010; Turnbull *et al*. 2012).

For these reasons, we also compared RGR at a common size (λ_s_), in seedlings by performing a species‐specific functional growth analysis with data from the growth analysis experiment. Growth functions were fitted to plots of logged total plant mass against time (plots shown in Fig. S2), using a four‐parameter logistic model, which allowed estimates of RGR at a common size for each species during the initial phase of growth (λ_s_), when RGR is expected to be highest (for full details of the fitting and RGR estimation see: Rose *et al*. 2009; Rees *et al*. 2010; Taylor *et al*. 2010; Turnbull *et al*. 2012). For this analysis, the common size used was the log of the minimum seedling mass (mg) for the largest species, which for grasses corresponded to 42·1 mg and for legumes was 64·7 mg. These sizes were selected as all species occur at these sizes and resource limitation should be minimal.

### Statistical Analyses

In order to determine how the various terms in eqn 1 influence the variance in yield, we used variance decomposition. To do this, we first write the parameter vector as θ = (*M*_s_, $\tilde{\lambda }$, *d*,* A*_r_, *c*), then the standard first‐order approximation to the variance is

(eqn 4) $$\text{Var}\left(Y\right)\approx \sum_{i}\sum_{j}\text{Cov}\left(\theta_{i},\theta_{j}\right)\frac{\partial Y}{\partial \theta_{i}}\frac{\partial Y}{\partial \theta_{j}},$$

where Cov is the covariance, Cov(θ_*i*_, θ_*i*_) = Var(θ_*i*_) the variance, and the subscripts *i* and *j* refer to different traits. This approach allows both the direct effects of variation in a trait, and indirect effects mediated through correlated changes in other traits (the covariance terms) to be assessed. For eqn 2, the same approach can be used with *θ* = (*M*_s_, *N*_s_, *N*_*i*_). The terms on the right‐hand side of eqn 4 define a square variance‐covariance matrix (Table S3) and so we can calculate the contribution to the variance of each trait by summing along the rows and dividing by the total, see Rees *et al*. (2010) for more details. Note the approach differs from that used in Rees *et al*. (2010) as yield (eqn 1) cannot be expressed as the sum of its components, and so we have to approximate the variance in yield using eqn 4 (more detail in Appendix S1).

Data from Yield Experiments 1 and 2 were then analysed in a phylogenetic context using r (R Core Team 2014). Data sets of plastid markers assembled previously for the grasses and legumes (Preece *et al*. 2015) were combined, and a tree including both groups (Fig. S3) was inferred with BEAST (Drummond & Rambaut 2007) as previously described (Preece *et al*. 2015). We used generalized least squares, using the pgls function in the caper package (Orme *et al*. 2013), to test for differences in species means. The difference in plant traits between crops and their progenitors was tested as a fixed effect, with models specified as follows: mod <‐ pgls(ln.yield ~ status, data = dat, lambda = ‘ML’).

Two other analyses using linear mixed effect models were performed in order to confirm the results of the pgls analysis. These were done with the lmekin function in the coxme package (Therneau, 2015) and the lme function in the nlme package (Pinheiro *et al*. 2014). For the lmekin analyses, the random effects fitted were block nested in experiment, and species, for example mod <‐ lmekin(ln.yield.g ~ status + (1|species) + (1|experiment/block), data = data.f, varlist = list(list(spp.var,var.cov.tree))). The species random effect included a phylogenetic component, and a between‐species component unrelated to phylogeny. For the lme analyses, the random effects fitted were accession, nested in crop, nested in family nested in block (and experiment where relevant), for example mod <‐ lme(ln.yield.g ~ status, random = ~1|experiment/block/family/crop/acc, data = data.f). In the results section, we show effect sizes and *P*‐values from the most conservative analysis (the pgls analysis). Correlations between traits among all species were also tested using the same statistical methods. The results of these analyses (Tables S4 and S5) are consistent with the pgls analysis, with some minor differences for the lme analysis.

For the growth analysis experiment, size‐corrected RGR, (λ_s_), was calculated for each species, and a pgls model was used to compare crops and their progenitors, similar to the other plant traits. Natural log transformations were applied to all variables except $\tilde{\lambda }$, λ_s_, *d*,* A*_r_ and *c*, and all comparisons were tested at the 0·05 significance level.

## Results

### Which Traits are Important for Determining Yield?

Overall, when considering eqn 1 for all species, variation in the mean individual mass of seeds sown (*M*_s_) made the greatest contribution to variation in total seed yield (*Y*) followed by variation in mean RGR ($\tilde{\lambda }$) (Table 2). The negative effect of variation in $\tilde{\lambda }$ occurs because this trait negatively covaries with *M*_s_, the growth period (*d*) and the allocation to reproductive biomass (*A*_r_). Hence, the positive effect of faster growth on yield is more than offset by reductions in *M*_s_, *d* and *A*_r_. When considering cereals in isolation, after *M*_s_, the proportion of chaff (*c*) is the second most important trait that contributes to variation in *Y*, although $\tilde{\lambda }$ and *d* were also fairly important. Pulses show the same pattern as all species considered together, so variation in yield was mainly due to variation in *M*_s_ and, to a lesser extent, $\tilde{\lambda }$.

**Table 2:** Contributions to the variance in total seed yield (*Y*) from variation in individual seed mass (*M*_s_), growth rate ($\tilde{\lambda }$), duration of growth (*d*), reproductive allocation (*A*_r_) and the proportion of chaff (*c*). The contribution to the variance of each trait is calculated by summing along the rows of the variance‐covariance matrix (Table S3) and dividing by the total. Note that because the contribution values include covariance terms, negative contributions can arise from negative covariance with other traits

	Individual seed mass (*M*_s_)	Growth rate ($\tilde{\lambda }$)	Duration of growth (*d*)	Reproductive allocation (*A*_r_)	Chaff (*c*)
All species	1·31	−0·38	−0·02	0·05	0·04
Cereals	0·61	0·19	−0·27	0·09	0·38
Pulses	1·32	−0·48	0·06	0·04	0·07

When considering eqn 2 for all species together, variation in individual seed mass, *M*_s_ (this time measured at harvest), is again the largest contributor to variation in *Y*, with seed number much less important. This result is mirrored for pulses analysed separately. However, for cereals, a different pattern is revealed by the additional trait data for these species, which shows that variation in seed number and infructescence number per plant is also important (Table 3).

**Table 3:** Contributions to the variance in total seed yield (*Y*) from variation in individual seed mass (*M*_s_), and total seed number, subdivided into seed number per infructescence (*N*_s_) and infructescence number per plant (*N*_i_) for the analysis of the cereals. The contribution to the variance of each trait is calculated by summing along the rows of the variance–covariance matrix (Table S3) and dividing by the total. Note that because the contribution values include covariance terms, negative contributions can arise from negative covariance with other traits

	Individual seed mass (*M*_s_)	Total seed number	Seed number per infructescence (*N*_s_)	Infructescence number per plant (*N*_i_)
All species	1·09	−0·09		
Cereals	0·48		0·31	0·21
Pulses	0·87	0·13		

### Which Traits Differ between Crops and their Progenitors?

**Figure 1:**
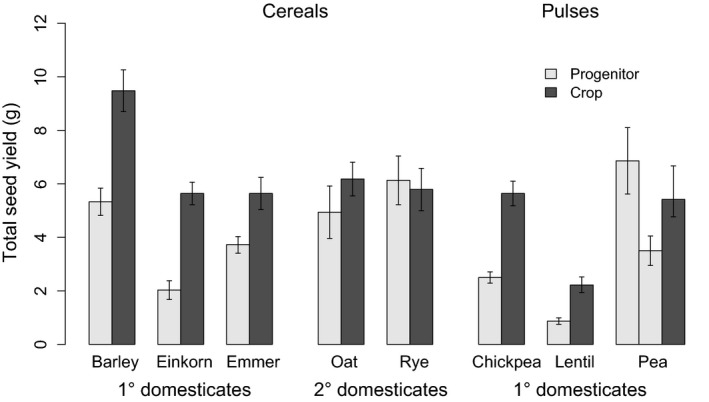
Total seed yield (g) of cereal and pulse crops and their progenitors. The pea progenitors are shown in the order *Pisum sativum* subsp. *elatius* and then *P. sativum* subsp. *elatius* var. *pumilio*, and this is the same in subsequent figures. Total seed yield is shown as the mean mass of grain harvested from each plant, combining the data from 2011 and 2013. In this figure, and subsequent figures, mean values are calculated from the raw data, rather than the fitted model. Crops are higher yielding (*P *<* *0·001), although this pattern is not present in cereal secondary domesticates.

**Figure 2:**
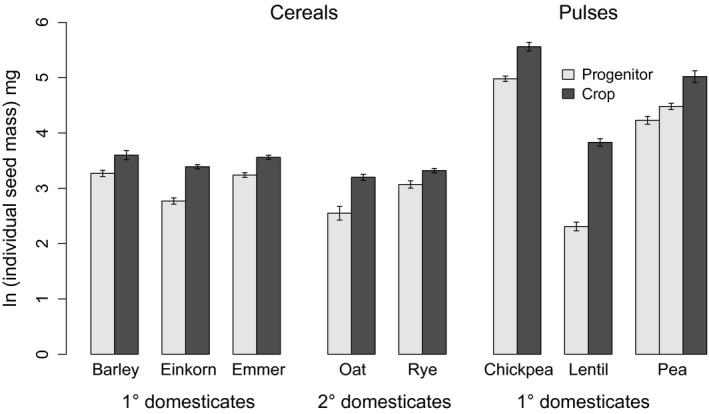
Individual seed mass (mg) of cereal and pulse crops and their progenitors. Seed mass is shown as the natural log of the mean mass of an individual grain, combining the data for the seeds that were sown in 2011 and 2013. Crops are larger seeded (*P *<* *0·0001).

**Figure 3:**
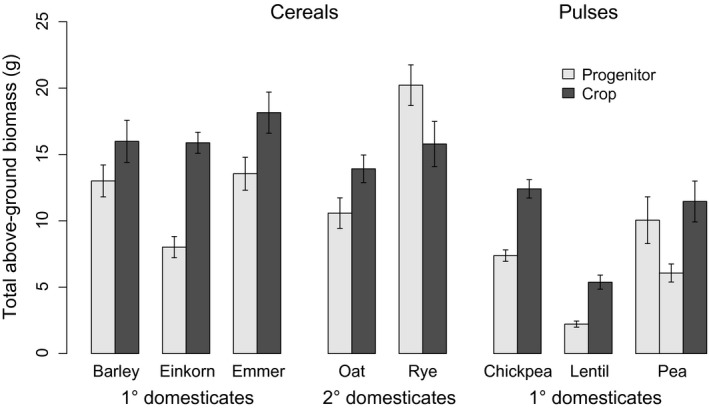
Total above‐ground biomass (g) of cereal and pulse crops and their progenitors, combining the data from 2011 and 2013. Crops in both families have greater biomass (*P *<* *0·001), with the exception of rye which shows the opposite pattern.

**Figure 4:**
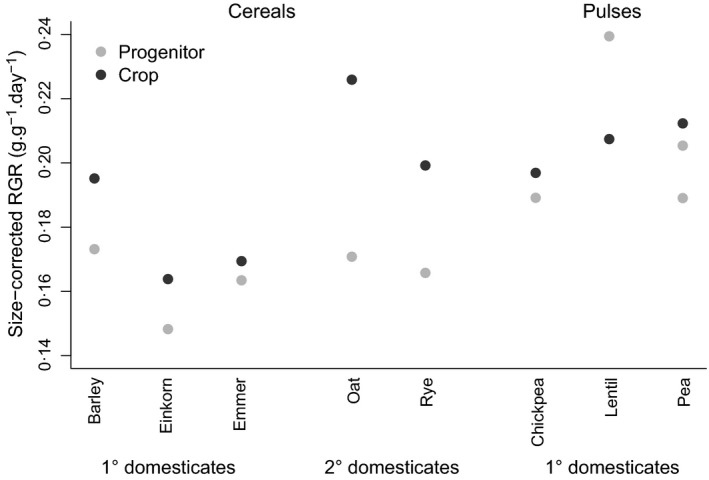
Size‐corrected relative growth rate (g g^−1^ day^−1^) for the cereal and pulse crops and their progenitors and crops. The mean values for each species are plotted. There is no significant difference between crops and their progenitors.

The comparison of crops with their progenitors showed that crops have significantly larger total seed yield (1·5× larger, 95% CIs [1·1, 2·1], *P* < 0·05), in support of our overall hypothesis (Fig. 1). There are differences among crop species, and notably, the effects of domestication on secondary cereal domesticates appear small, with no significant difference in total seed yield for either oats or rye (Fig. 1a). Crops also had greater individual sown seed mass (1·9× larger, 95% CIs [1·4, 2·5], *P* < 0·0001) (Fig. 2) and greater total above‐ground biomass (1·4× larger, 95% CIs [1·2, 1·7], *P* < 0·05) (Fig. 3). There was, however, no difference in the duration of growth (*d*), allocation to reproductive biomass (*A*_r_) or height (Table 4). Growth rate did not differ between crops and progenitors, either when calculated as average RGR in the yield experiments ($\tilde{\lambda }$) or as size‐corrected RGR in the separate growth analysis (λ_s_), using the functional approach at a common size (Fig. 4).

**Table 4:** Effect of domestication on reproductive and vegetative traits. All analyses consider cereals and pulses in combination unless otherwise stated. A phylogenetic generalized least squares (pgls) analysis was carried out with domestication status as a fixed effect

Trait	Effect of domestication	*P*	*F*_df_
*Y* – total seed yield	Crops have 1·5× higher yields	<0·05	8·0_1,15_
*M*_s_ – individual seed mass	Crops 1·9× larger	<0·0001	17·4_1,15_
*d* – duration of growth		NS	
*A*_r_ – % reproductive mass		NS	
*c* – proportion of chaff	Crops have 38% less chaff	<0·01	12·6_1,15_
Total above‐ground biomass	Crops have 1·4× greater biomass	<0·05	7·1_1,15_
$\tilde{\lambda }$‐relative growth rate		NS	
λ_s_ – size‐corrected growth rate		NS	
Total seeds per plant		NS	
Height		NS	
Infructescence mass	Crops have 2·1× larger spikes/pods	<0·001	27·4_1,15_
Cereals only			
*N*_s_ – number seeds per infructescence	Cereal crops have 1·3× more seeds	<0·01	13·5_1,8_
*N*_i_ – number of infructescences		NS	

NS, non‐significant.

All traits were natural log‐transformed except *d*,* Ar*,* c* and $\tilde{\lambda }.$ *P*‐values < 0·05 are reported.

**Figure 5:**
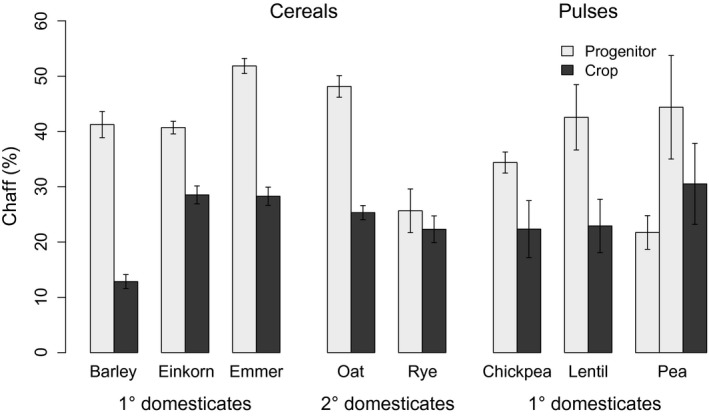
Percentage chaff or pod material in crops and their progenitors, calculated as (chaff/(chaff + grain)) × 100. Crops have lower % chaff or pod material than their progenitors (*P *<* *0·01). Note that there is no difference between progenitors and crops for rye and pea.

Crops had a lower proportion of chaff making up their reproductive biomass (24·2%) than their progenitors (39·0%) (38% less, 95% CIs [18·0, 58·2], *P* < 0·01) (Fig. 5). Cereals also had a greater number of seeds per spike (1·3× greater, 95% CIs [1·2, 1·6], *P* < 0·01) and greater spike mass (1·7× greater, 95% CIs [1·2, 2·5], *P *<* *0·05). Total seed number per plant did not differ between crops and progenitors. Seed number per gram of plant biomass was also calculated and did not differ between crops and their progenitors. Mean values of all measured traits are shown for each species in the Supporting Information (Table S6).

Above‐ground biomass was strongly positively correlated with total seed yield across all species (*P *<* *0·0001, *R* ^2^ = 0·84), with no interaction with domestication status (crop versus progenitor) (Fig. S4). Individual seed mass at sowing was positively correlated with individual seed mass at harvest, (*P *<* *0·0001, *R* ^2^ = 0·97) total seed yield (*P *<* *0·001, *R* ^2^ = 0·54) and above‐ground biomass (*P *<* *0·001, *R* ^2^ = 0·59) when compared across species (Figs S1, S5 and S6).

## Discussion

This study examined the components of yield that distinguish crop landraces from their wild progenitors. Total seed yield was greater for crops than their wild relatives, and we also looked at the specific ways in which the components of yield differed consistently between domesticated and wild plants. Individual seed mass was a key trait, determining total seed yield, and in cereals, the production of more seeds per spike and less chaff was also important.

The processes of cultivation and domestication may represent a continuum (Gepts 2012), in which the species taken into cultivation depend on particular plant traits, and then, the way in which they are domesticated depends on other plant traits. This study follows previous work that investigated traits common to crop progenitors, in order to understand why some types of plants were domesticated instead of others (Preece *et al*. 2015). Here, a similar experimental approach is used, with the focus on a later stage of the same process. Together these two studies indicate that the high yield of crops arose later in the cultivation–domestication continuum and was not a trait already present in crop progenitors. Large seed size is a key trait in Fertile Crescent crops because these species were larger seeded in the cultivation stage (progenitors larger than other wild species), and then, there was a further increase in the domestication stage (landraces larger than their progenitors). However, bigger seed size alone is not enough to increase yields (as shown by the previous work on crop progenitors), and this new study also supports the inclusion of widening the plant functional traits that we associate with the domestication process.

The study is novel in looking at whether the domestication syndrome can be expanded to include additional traits common to multiple crop species. Specifically, it showed how traits related to growth and allocation differed between the species, including the ways in which some of these traits covaried. Overall, we demonstrate the importance of large size for both cereal and pulse crops, both at the scale of individual seeds and the whole plant.

### Growth and Allocation

The first approach for calculating total seed yield, using traits relating to growth and allocation (eqn 1) predicts that greater yield should arise from any increase in plant size (a function of initial seed size, growth rate and the duration of growth), greater allocation to reproductive tissues, or a decrease in allocation to chaff. Domesticated species had greater biomass than their progenitors in agreement with previous work showing that landraces are larger than their wild progenitors (Evans 1993; Milla *et al*. 2014). However, whether these larger sizes were the consequence of different growth strategies remains uncertain. Growth rates can be calculated in different ways, and the use of classical RGR has been questioned in situations where initial plant sizes are very different, leading to the development of methods accounting for size (Turnbull *et al*. 2008). In this study, we found no differences in growth rate calculated for plants at a common size. Instead, the final size advantage appears mostly to come from a seed size that was initially larger, that is a ‘head‐start’. Overall, we found no evidence for an effect of domestication on growth rates.

During modern crop breeding programmes, there has been a focus on increasing allocation to reproductive biomass, and in particular the harvest index (Hay 1995; Fischer & Edmeades 2010), including the breeding of semi‐dwarf varieties of modern cereals (Sakamoto & Matsuoka 2004). The landraces of crops in our study had greater overall biomass than their wild progenitors, but did not differ in allocation patterns between reproductive and vegetative biomass, with no differences in harvest index or final height. No difference in biomass allocation between wild progenitors, landraces and modern cultivars of wheat has previously been suggested (Damisch & Wiberg 1991). Therefore, without any difference in allocation to reproductive tissue, total seed yield was increased as a result of the larger initial and final plant sizes of crops. Crops also had a lower proportion of chaff or pod material compared with their wild progenitors, further augmenting the yield.

It is important to note that our results apply to plants grown individually, and that plant size may be affected by competition (Gurevitch *et al*. 1990). In general, as plants are grown at higher densities, biomass and yield per unit area increase up to a threshold level, after which values remain more or less constant. The occurrence of this ‘constant final yield’ happens when plants experience intraspecific competition for resources, and individual size and yield cannot reach maximum levels (Donald 1951; Weiner & Freckleton 2010). The amount of competition and relative competitive ability of different species is therefore an important factor in determining yield per unit area. Another consideration is that high yields per individual do not necessarily correspond to high yield per hectare. Individuals that produce a high seed output often do this by being excellent competitors, but high individual competitive ability can lead to low overall biomass of a stand, as there a few ‘winners’ and many ‘losers’ (Anten & Vermeulen 2016). It has been suggested that ideal crops should be weak competitors to keep intraspecific competition at a minimum (Donald 1968; Zhang, Sun & Jiang 1999). However, crop progenitors may have been better competitors than other wild species, demonstrating some traits that may be undesirable in crops growing at high density (e.g. tall stature and large leaf area), so the species in our study may be particularly prone to not maximizing community performance (Anten & Vermeulen 2016).

Whilst care should therefore be taken when considering the importance of plant size, our results allow us to compare the different species under optimum conditions of no or minimal competition, as would occur when sown at low densities. We also do not know at what densities the plants were grown during the early phases of domestication, and it may well be that plants were grown at close to optimal conditions.

### Seed Mass and Number

The second way of calculating total seed yield (eqn 2) predicts that yield can increase as a function of individual seed mass, the number of seeds per infructescence or the number of infructescences per plant. All of the crops in this study had greater individual seed mass than their progenitors, which would, in the absence of trade‐offs, lead to greater yields. However, across species, larger individual seed size tends to be negatively correlated with seed number, which stabilizes yield (Leishman 2001; Coomes & Grubb 2003; Sadras 2007). In fact, a previous study of yield‐related traits found that these large‐seeded cereal crop progenitors have significantly fewer seeds per plant than other Fertile Crescent grasses (Preece *et al*. 2015). Seed size‐number trade‐offs have been linked with competition–colonization trade‐offs, with smaller seeds having greater dispersal ability (Turnbull, Rees & Crawley 1999), and tolerance–fecundity trade‐offs, whereby large seeds have an advantage of higher tolerance of stresses (Muller‐Landau 2010).

In this study, the large‐seeded cereal crops had a greater number of seeds per spike but we found no evidence of any reduction in seed number per plant, such that domesticated and wild plants had similar numbers of seeds. The lack of a negative relationship between seed size and number may be a consequence of yield being largely determined by variation in seed mass, which in turn implies that the amount of resources captured increases with seed mass. When large‐seeded species are much better at capturing resources, and become larger adult plants, then positive relationships between seed mass and number are possible (Venable 1992). Also, crops had a lower proportion of chaff than their progenitors, indicating a change in resource allocation between grain and chaff and possibly helping to explain the lack of a seed size/number trade‐off.

We also did not see a conclusive reduction in seed number per gram of plant biomass, at least when analysed in a phylogenetic context (see Table S4). This is important as it rules out the possibility that the seed size‐number trade‐off was absent because larger crop plants acquired more resources than their wild progenitors, enabling them to produce a similar number of larger seeds. The fact we did not see a reduction in seed number is important for understanding how the yield advantage of crops may have arisen through unconscious selection; cultivation and harvesting of plants relaxes selection on seed size for the purpose of dispersal (i.e. it allows larger seed size) (Brown *et al*. 2009). In a genetically diverse population of a crop progenitor under cultivation, larger seeded genotypes might therefore gain a selective advantage under competition in dense stands, once selection for dispersal and dormancy is relaxed. By also having the same or a higher number of seeds, they would be able to increase their numerical advantage within the population. This two‐pronged strategy, when found in combination with indehiscent seeds, may provide a mechanism that enables species to produce high seed yields that would be easily harvestable, and thus be successful crops. Alternatively, people could have bred from the plants with the largest ears, which had both larger seeds and more seeds.

Interestingly, if we look at the comparisons of total seed yield between the cereal crops and their corresponding progenitors, it is noticeable that only the three primary domesticates (barley, einkorn and emmer wheat) show a significant yield advantage over their wild relatives. Our data suggest that the later domestication of oats and rye resulted in smaller increases in yield, which is interesting because these species had been under selection as agricultural weeds in cultivated habitats since the origins of agriculture (Vavilov 1926; Zohary, Hopf & Weiss 2012). The absence of a domestication effect in these species may therefore arise because their wild progenitors had already been under similar selection pressures to barley, einkorn and emmer for several thousand years.

## Conclusions

Overall, these experiments demonstrate the general importance of size throughout the life cycle of a crop, whereby under optimum conditions large seeds grow into large plants, which in turn produce high yields. Reproductive organs also change, such that a higher proportion of reproductive biomass is edible grain, and seed number is not negatively impacted by the increases in individual seed mass. The combination of these traits, together with a mutation for indehiscence, resulted in plants that were successful food resources for traditional farmers. It is therefore important to broaden the domestication syndrome to recognize the importance of both individual seed size and plant size as components of the domestication syndrome for cereals and pulses from the Fertile Crescent.

## Data accessibility

Data for this paper have been archived in figshare: https://dx.doi.org/10.6084/m9.figshare.3790578.v1.

## Supporting information


**Lay Summary**

**Fig. S1.** Plot of natural‐logged sown seed mass and harvested seed mass in Yield Experiment 1.
**Fig. S2**. Plots of natural‐logged biomass over time for species in the growth analysis experiment.

**Fig. S3.** Phylogeny for the 17 species used in our experiments.

**Fig. S4.** Correlation between ln(total seed yield) and ln(total above‐ground biomass) for the cereal and pulse species.
**Fig. S5.** Correlation between ln(total seed yield) and ln(individual seed mass) for the cereal and pulse species.
**Fig. S6.** Correlation between ln(total above‐ground biomass) and ln(individual seed mass) for the cereal and pulse species.
**Appendix S1.** A brief description of the variance approximation used for the variance decomposition analysis.

**Table S1**. List of grass accessions used in study.
**Table S2.** List of legume accessions used in study.

**Table S3.** Variance‐covariance matrices for eqns 1 and 2.
**Table S4.** Comparison of three statistical methods used to analyse differences between crops and their progenitors.
**Table S5.** Comparison of three statistical methods used to analyse between‐species correlations.

**Table S6.** Species means of traits measured in the yield experiments.
