# Supplementary material for: How did the domestication of Fertile Crescent grain crops increase their yields?
Source: Funct Ecol. 2016 Oct 3;31(2):387–97. doi: 10.1111/1365-2435.12760 (PMC5324541; doi:10.1111/1365-2435.12760)
Supplement: Supplementary file 1 — Lay Summary [file FEC-31-387-s001.pdf]

Functional Ecology

British Ecological Society

New insights into the origins of agriculture: How did the domestication of Fertile Crescent grain crops increase their yields?

Catherine Preece, Alexandra Livarda, Pascal-Antoine Christin, Michael Wallace, Gemma Martin, Michael Charles, Glynis Jones, Mark Rees and Colin P. Osborne

Humans began farming the land 10,000 years ago, leading to an agricultural revolution, which had big impacts on human societies. The plants that we now use as crops have changed a lot since the first farmers started growing them. During the process of domestication, these plants changed in appearance and the amount of food that they produce increased. However, we do not fully understand how these changes happened. A deeper understanding of the origins of agriculture may prove useful for improving food production today.

We grew traditional versions of crops including wheat, barley, lentils and peas, and compared them with their wild ancestors, to see how they have changed through domestication, measuring a much wider range of characteristics than ever before. We found that crops are able to produce more food for us because of three important characteristics: they grow into bigger plants, they have larger seeds and they contain less non-edible material. Crops are also able to produce the same number of seeds as their wild ancestors, despite the seeds being bigger. This is important because normally plants must choose between producing a few big seeds or lots of small seeds, but this does not seem to apply so much to these plants.

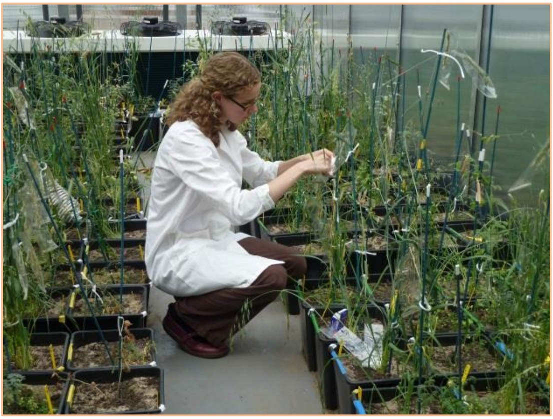

The lead author (C. Preece) working in the greenhouse growing crops and their wild ancestors. Photo credit – Catherine Preece.

Today we are seeing increasing pressure on global food production, and crop breeders are taking an increasing interest in traditional crops as a source of useful traits that may help to increase yields or increase resilience to climate change. Our work should help in this process by providing new insights into the process of crop domestication, which may lead to innovations in modern agriculture.
