# Supplementary material for: How did the domestication of Fertile Crescent grain crops increase their yields?
Source: Funct Ecol. 2016 Oct 3;31(2):387–97. doi: 10.1111/1365-2435.12760 (PMC5324541; doi:10.1111/1365-2435.12760)

Fig. S1 Plot of natural-logged sown seed mass and harvested seed mass for all species in the Yield experiment 1. Each point shows a different species. $R^2 = 0.97$, $P < 0.0001$. The blue line shows the 1:1 line.

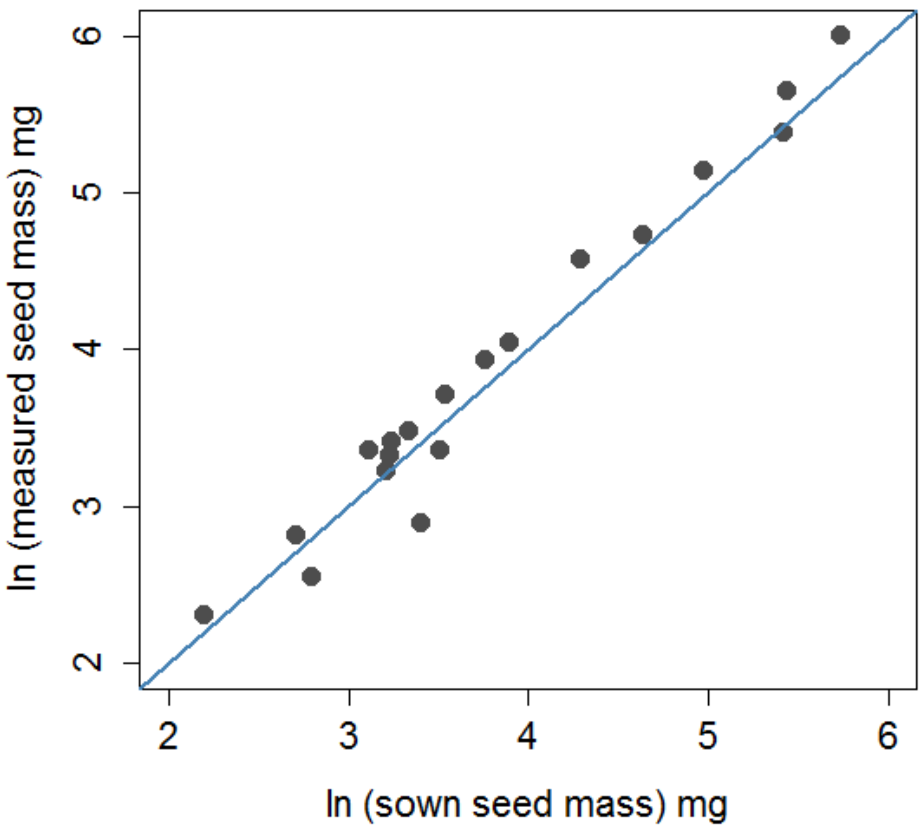

Fig. S2 Plots of fitted curves for logged (ln) biomass over time for species in the growth analysis experiment. Grey line shows model mean and blue lines show fit for each species.

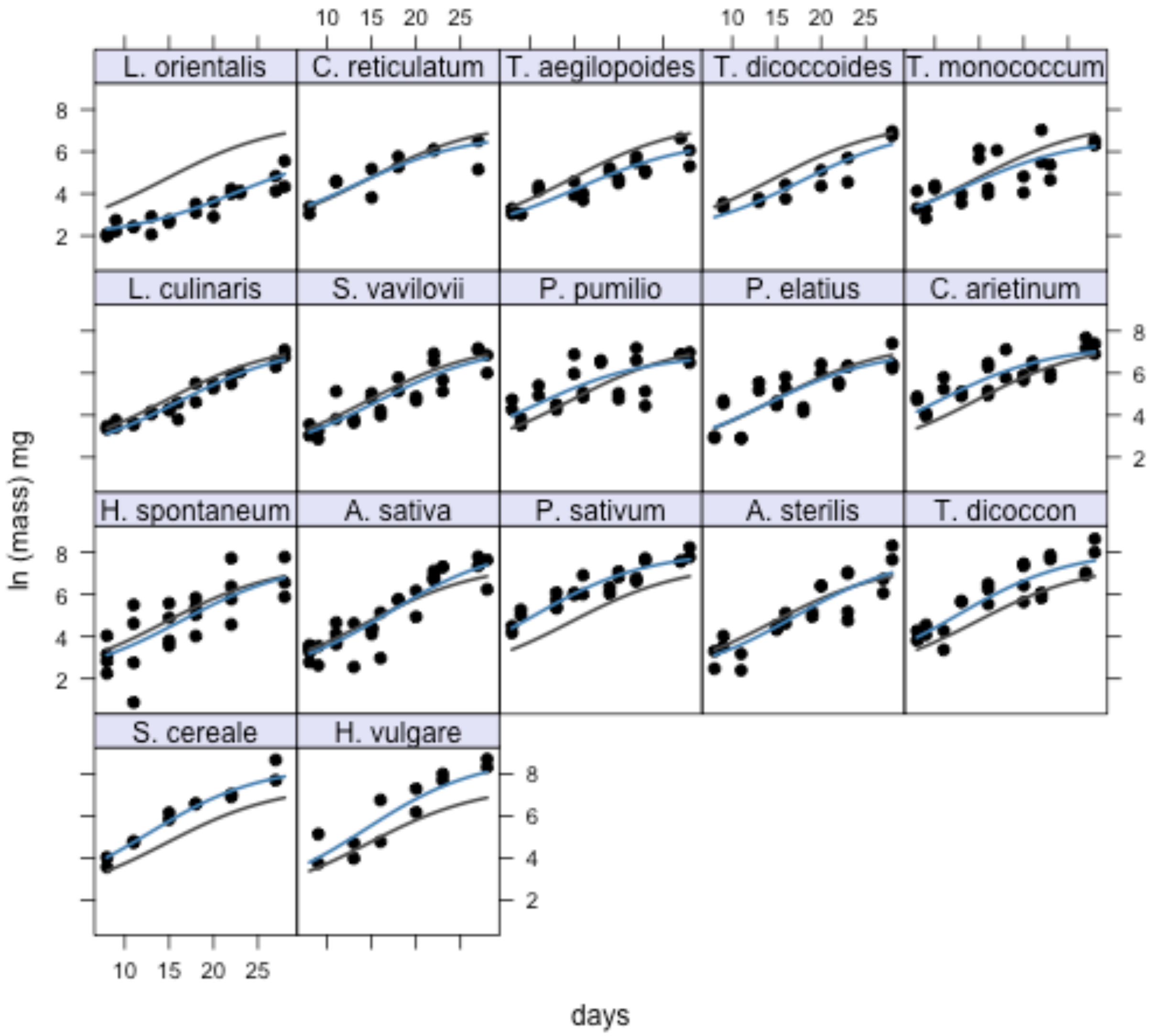

Supplement: Supplementary file 2 — Fig. S1. Plot of natural‐logged sown seed mass and harvested seed mass in Yield Experiment 1. Fig. S2. Plots of natural‐logged biomass over time for species in the growth analysis experiment. [file FEC-31-387-s002.pdf]
