# Supplementary material for: How did the domestication of Fertile Crescent grain crops increase their yields?
Source: Funct Ecol. 2016 Oct 3;31(2):387–97. doi: 10.1111/1365-2435.12760 (PMC5324541; doi:10.1111/1365-2435.12760)

Fig. S3 Phylogeny for the 17 species used in our experiments, based on the two plastid markers *ndhF* and *trnKmatK*.

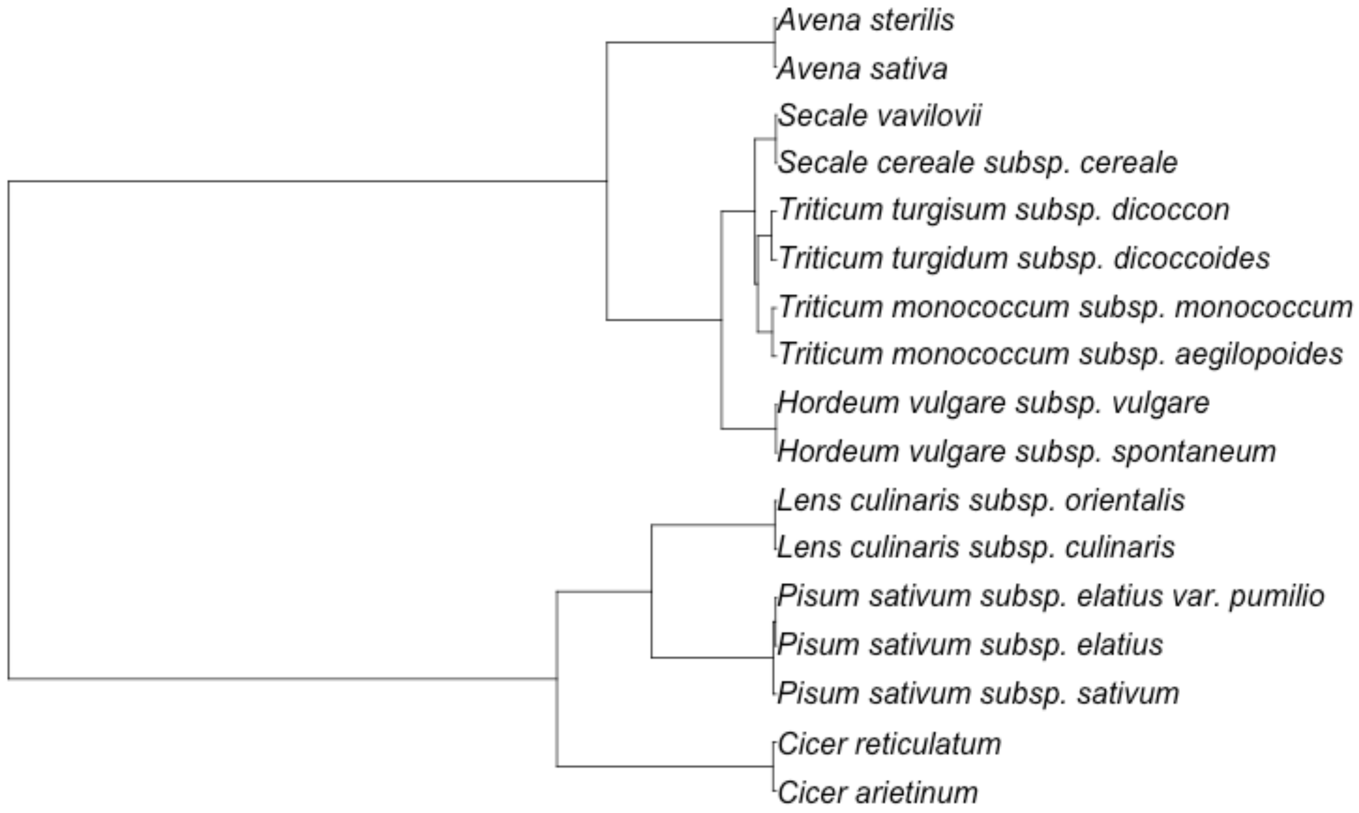

Supplement: Supplementary file 3 — Fig. S3. Phylogeny for the 17 species used in our experiments. [file FEC-31-387-s003.pdf]
