# Supplementary material for: How did the domestication of Fertile Crescent grain crops increase their yields?
Source: Funct Ecol. 2016 Oct 3;31(2):387–97. doi: 10.1111/1365-2435.12760 (PMC5324541; doi:10.1111/1365-2435.12760)
Supplement: Supplementary file 4 — Fig. S4. Correlation between ln(total seed yield) and ln(total above‐ground biomass) for the cereal and pulse species. Fig. S5. Correlation between ln(total seed yield) and ln(individual seed mass) for the cereal and pulse species. Fig. S6. Correlation between ln(total above‐ground biomass) and ln(individual seed mass) for the cereal and pulse species. Appendix S1. A brief description of the variance approximation used for the variance decomposition analysis. [file FEC-31-387-s004.pdf]

Fig. S4 Correlation between ln(total seed yield) and ln(total aboveground biomass) for the cereal and pulse species. The mean values of the raw data are plotted. $R^2 = 0.86$, $P < 0.0001$.

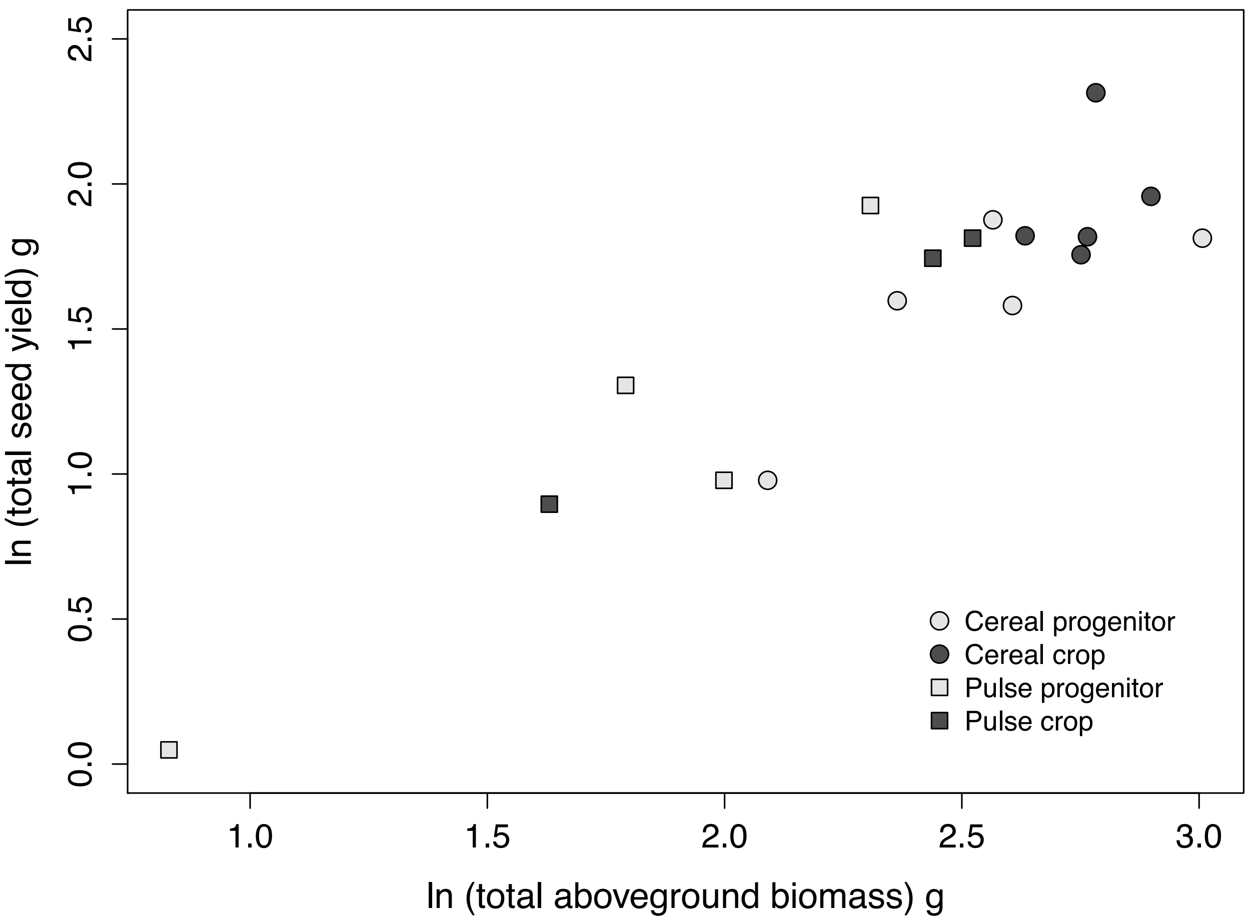

Fig. S5 Correlation between ln(total seed yield) and ln(individual seed mass) for the cereal and pulse species. The mean values of the raw data are plotted. R^2 = 0.54, P < 0.001.

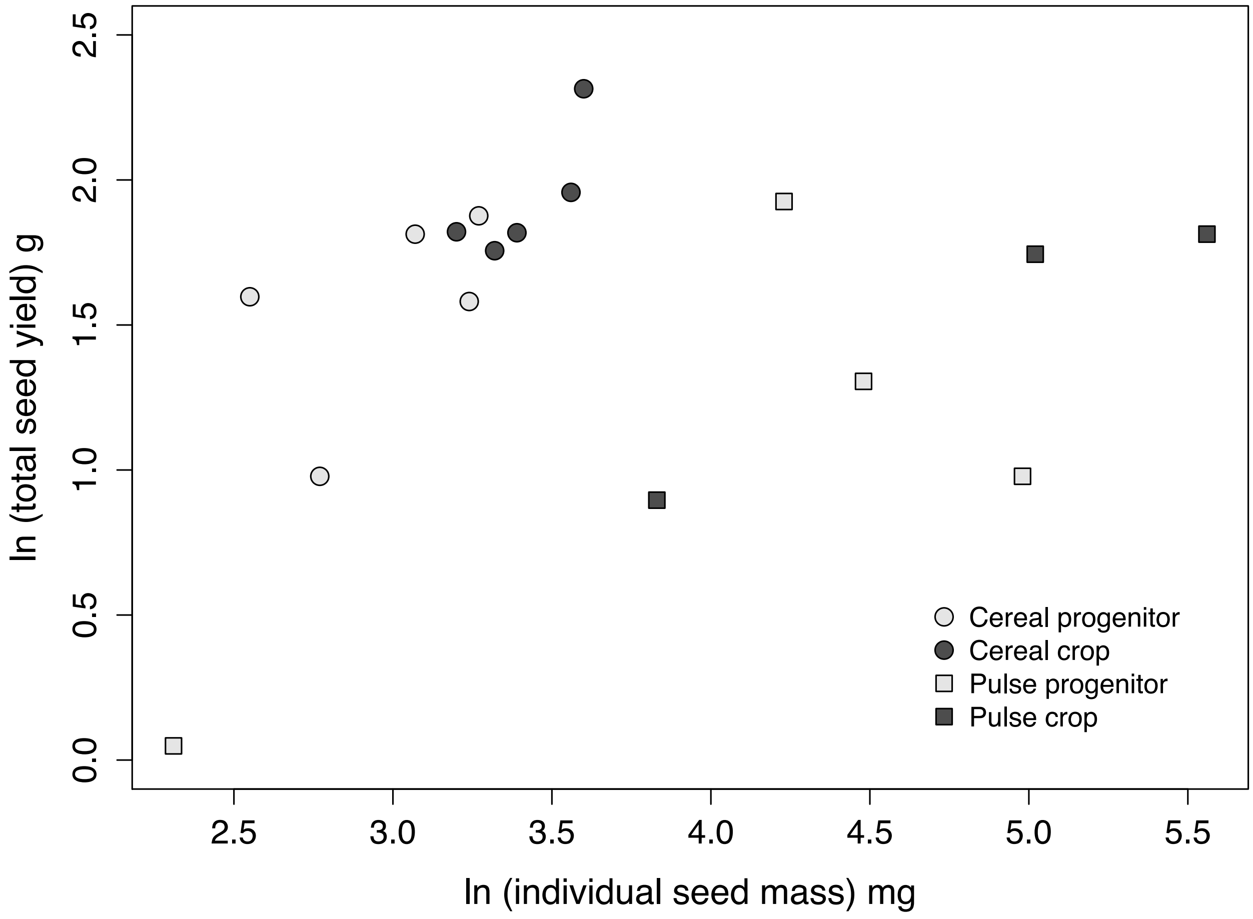

Fig. S6 Correlation between ln(total aboveground biomass) and ln(individual seed mass) for the cereal and pulse species. The mean values of the raw data are plotted. $R^2 = 0.60$, $P < 0.001$.

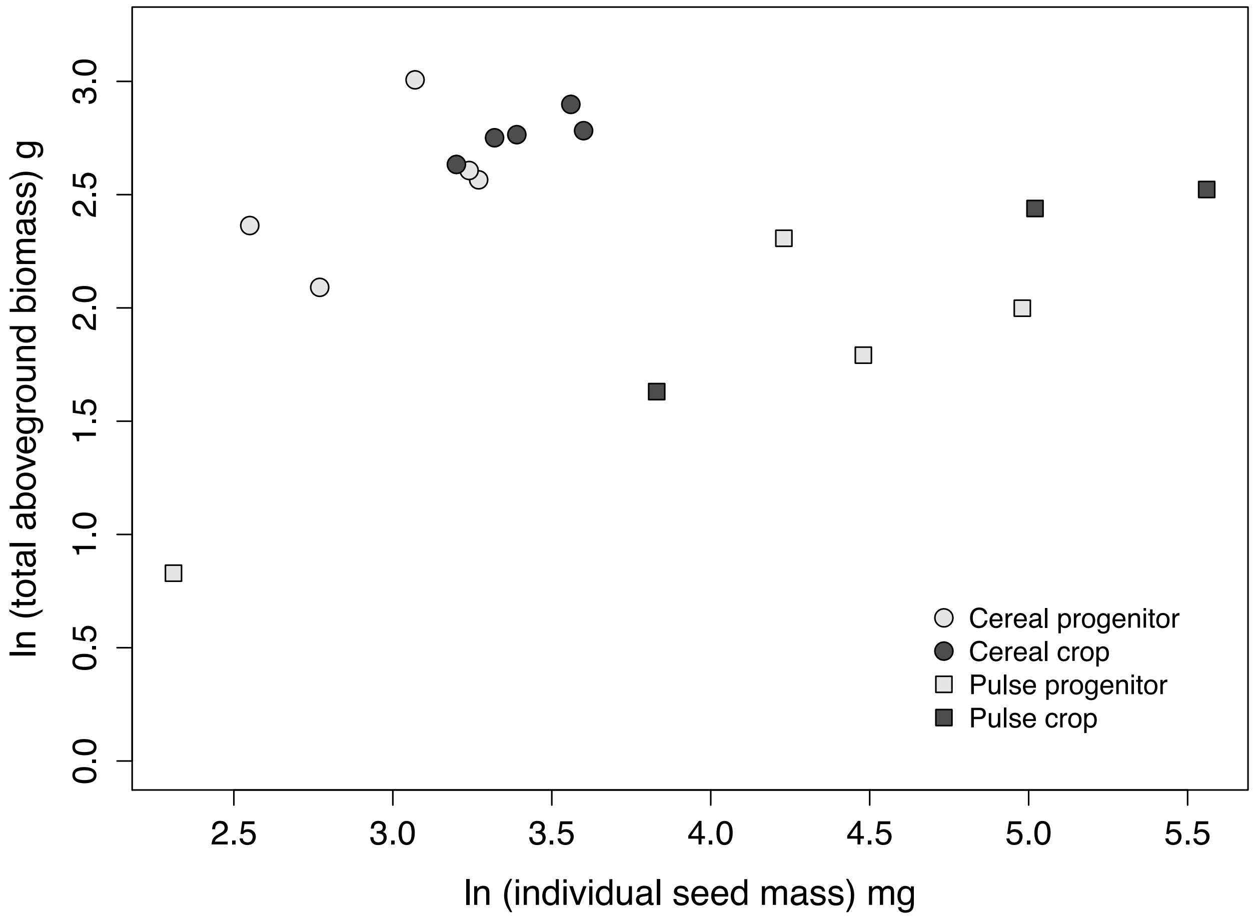

Appendix S1 A brief description of the variance approximation used for the variance decomposition analysis.

Assume we want to approximate the variance of some function $f(x,y)$ where x and y are random variables with means μ_x, μ_y and the variances and covariance given by $V(x), V(y)$ and $C(x,y)$ respectively.

The standard first order Taylor approximation of $f(x,y)$ evaluated at the means is

$$f(x,y) \approx f(\mu_x, \mu_y) + \frac{\partial f(x,y)}{\partial x}(x - \mu_x) + \frac{\partial f(x,y)}{\partial y}(y - \mu_y)$$

Taking variances of this expression we get

$$V(f(x,y)) \approx \left(\frac{\partial f(x,y)}{\partial x}\right)^2 V(x) + \left(\frac{\partial f(x,y)}{\partial y}\right)^2 V(y) + 2\frac{\partial f(x,y)}{\partial x}\frac{\partial f(x,y)}{\partial y}C(x,y)$$

where all the partial derivatives are evaluated at μ_x, μ_y. This is the standard delta method approximation to the variance of a function.
