# Supplementary material for: How did the domestication of Fertile Crescent grain crops increase their yields?
Source: Funct Ecol. 2016 Oct 3;31(2):387–97. doi: 10.1111/1365-2435.12760 (PMC5324541; doi:10.1111/1365-2435.12760)
Supplement: Supplementary file 5 — Table S1. List of grass accessions used in study. Table S2. List of legume accessions used in study. [file FEC-31-387-s005.pdf]

Supporting Information for C. Preece *et al*., 2016, "How did the domestication of Fertile Crescent grain crops increase their yields?"

Table S1 List of grass accessions used, including the seed bank they were obtained from, country of origin and mean individual seed mass in mg (± 1SE). Seed banks used were the USDA Germplasm Resources Information System (GRIN), IPK Gatersleben Genebank (IPK) and John Innes Centre Germplasm Resources Unit (JIC).

Species	Group	Seed bank	Accession numbers	Country of origin	Individual seed mass mg (± 1SE)	Experiment
Avena sativa L.	Landrace	GRIN	PI 168072	Turkey	30.21 ± 0.95	2011 yield and RGR
		GRIN	PI 173583	Turkey	22.08 ± 0.59	2011 yield and RGR
Avena sterilis L.	Progenitor	GRIN	PI 326955	Israel	11.18 ± 0.59	2011 yield
		GRIN	PI 134251	Afghanistan	22.32 ± 0.19	2011 yield
		GRIN	PI 309424	Israel	13.64 ± 1.31	2011 yield
		GRIN	PI 309527	Israel	11.52 ± 0.72	RGR
		GRIN	PI 220372	Afghanistan	16.05 ± 0.72	RGR
Hordeum vulgare L. subsp. *spontaneum* (K. Koch) Thell.	Progenitor	GRIN	PI 282628	Israel	19.37 ± 1.34	2011 yield
		GRIN	PI 466114	Syria	26.76 ± 1.08	2011 yield
		GRIN	PI 236387	Syria	28.67 ± 0.74	2013 yield
		GRIN	PI 354944	Israel	33.27 ± 2.30	2013 yield
		GRIN	HOR2688	Iran	25.45 ± 2.18	2013 yield
		GRIN	PI 282656	Israel	12.86 ± 0.79	RGR
		GRIN	PI 466126	Syria	28.75 ± 0.88	RGR

Hordeum vulgare L. subsp. *vulgare*	Landrace	GRIN	Ciho 1464	Iraq	44.99 ± 0.95	2011 yield and RGR
		GRIN	Ciho 1465	Iraq	26.43 ± 1.06	2011 yield
		JIC	18208	Iran	28.39 ± 3.28	2013 yield
		JIC	3604	Israel	49.29 ± 3.79	2013 yield
		JIC	20153	Syria	42.97 ± 5.80	2013 yield
Secale cereale L. subsp. *cereale*	Landrace	GRIN	PI 168130	Turkey	25.71 ± 0.60	2011 yield
		GRIN	PI 357058	Turkey	29.27 ± 0.24	2011 yield and RGR
Secale vavilovii Grossh.	Progenitor	GRIN	PI 284842	Hungary	28.81 ± 0.72	2011 yield and RGR
		GRIN	PI 573649	Afghanistan	21.74 ± 0.63	2011 yield and RGR
Triticum monococcum L. subsp. *aegilopoides* (Link) Thell.	Progenitor	GRIN	PI 427452	Turkey	14.51 ± 1.39	2011 yield
		GRIN	PI 245726	Turkey	16.13 ± 2.25	2011 yield
		GRIN	PI 554577	Turkey	13.41 ± 2.43	2013 yield
		GRIN	PI 427997	Lebanon	14.97 ± 1.25	2013 yield
		IPK	TRI17105	Iran	20.26 ± 0.61	2013 yield
		GRIN	PI 352503	Iran	14.18 ± 0.66	RGR
		GRIN	PI 427697	Iraq	12.37 ± 0.99	RGR

Triticum monococcum L. subsp. *monococcum*	Landrace	GRIN	PI 428161	Turkey	25.71 ± 0.67	2011 yield and RGR
		GRIN	PI 167615	Turkey	31.16 ± 0.84	2011 yield
		GRIN	PI 427927	Iraq	27.51 ± 1.81	2013 yield
		JIC	1040052	Syria	33.46 ± 2.58	2013 yield
		JIC	1040041	Turkey	26.98 ± 1.78	2013 yield
		GRIN	PI 167526	Turkey	30.26 ± 1.21	RGR
Triticum turgidum L. subsp. *dicoccoides* (Körn. ex Asch. & Graebn.)Thell.	Progenitor	GRIN	PI 300989	Israel	27.28 ± 1.04	2011 yield
		GRIN	PI 428022	Turkey	23.23 ± 0.98	2011 yield
		GRIN	PI 352324	Lebanon	26.71 ± 1.09	2013 yield and RGR
		GRIN	PI 428017	Turkey	43.11 ± 4.33	2013 yield
		GRIN	PI 487242	Syria	27.55 ± 2.50	2013 yield
Triticum turgidum L. subsp. *dicoccon* (Schrank) Thell.	Landrace	GRIN	PI 470737	Turkey	35.31 ± 1.30	2011 yield
		GRIN	PI 470739	Turkey	32.51 ± 1.05	2011 yield and RGR
		GRIN	PI 182743	Turkey	50.24 ± 1.58	2013 yield
		GRIN	PI 624903	Iran	43.65 ± 1.65	2013 yield
		JIC	1070009	Iran	36.04 ± 0.64	2013 yield
		GRIN	PI 94626	Turkey	45.23 ± 1.74	RGR

Table S2 List of legume accessions used, including the seed bank they were obtained from, country of origin and mean individual seed mass in mg (± 1SE). Seed banks used were the USDA Germplasm Resources Information System (GRIN), and John Innes Centre Germplasm Resources Unit (JIC).

Species	Group	Seed bank	Accession	Country of origin	Mean seed mass mg (± 1SE)	Experiment
Cicer arietinum L.	Landrace	GRIN	PI 543059	Pakistan	153.9 ± 4.50	2011 yield and RGR
		GRIN	PI 339154	Turkey	320.0 ± 13.07	2011 & 2013 yield and RGR
		GRIN	PI 339186	Turkey	327.0 ± 23.29	2013 yield
Cicer reticulatum Ladiz.	Progenitor	GRIN	PI 489777	Turkey	127.2 ± 5.41	2011 & 2013 yield and RGR
		GRIN	PI 510656	Turkey	141.0 ± 4.51	2011 yield
		GRIN	PI 599092	Turkey	156.7 ± 15.32	2013 yield
Lens culinaris subsp. *culinaris*	Landrace	GRIN	PI 339294	Turkey	34.8 ± 0.58	2011 yield and RGR
		GRIN	PI 339263	Turkey	64.4 ± 1.00	2011 yield and RGR
		GRIN	PI 298026	Turkey	22.8 ± 2.27	2013 yield
		GRIN	PI 308609	Syria	61.9 ± 4.85	2013 yield
		GRIN	PI 308610	Syria	43.9 ± 7.53	2013 yield
Lens culinaris subsp. *orientalis* (Boiss.)	Progenitor	GRIN	PI 572371	Israel	8.2 ± 0.25	2011 & 2013 yield and RGR
		GRIN	PI 572379	Turkey	10.6 ± 0.24	2011 yield and RGR
		GRIN	PI 572374	Iran	19.0 ± 1.56	2013 yield
		GRIN	PI 572366	Turkey	8.7 ± 0.69	2013 yield

Pisum sativum L. subsp. *elatius* (Steven ex M. Bieb.) Asch. & Graebn.	Progenitor	GRIN	PI 344004	Turkey	62.0 ± 1.65	2011 yield and RGR
		GRIN	PI 343972	Turkey	87.7 ± 2.31	2011 yield and RGR
Pisum sativum L. subsp. *elatius* (Steven ex M. Bieb.) Asch. & Graebn. var. *pumilio* Meikle	Progenitor	GRIN	W6 2107	Turkey	120.7 ± 5.68	2011 yield
		GRIN	W6 12613	Turkey	76.4 ± 3.08	2011 yield and RGR
		GRIN	W6 2101	Turkey	70.3 ± 6.14	2013 yield
		JIC	3273	Israel	82.6 ± 4.79	2013 yield
		JIC	3274	Israel	72.9 ± 2.63	2013 yield
		GRIN	PI 15013	Egypt	132.5 ± 9.49	RGR
Pisum sativum L. subsp. *sativum*	Landrace	GRIN	PI 343986	Turkey	188.44 ± 4.90	2011 yield
		GRIN	PI 343987	Turkey	215.2 ± 6.59	2011 yield and RGR
		JIC	1843	Israel	165.1 ± 13.89	2013 yield
		JIC	3226	Syria	69.8 ± 2.95	2013 yield
		JIC	1073	Turkey	89.6 ± 8.86	2013 yield
		GRIN	PI 343985	Turkey	201.0 ± 9.47	RGR
