# Supplementary material for: How did the domestication of Fertile Crescent grain crops increase their yields?
Source: Funct Ecol. 2016 Oct 3;31(2):387–97. doi: 10.1111/1365-2435.12760 (PMC5324541; doi:10.1111/1365-2435.12760)
Supplement: Supplementary file 6 — Table S3. Variance‐covariance matrices for eqns 1 and 2. Table S4. Comparison of three statistical methods used to analyse differences between crops and their progenitors. Table S5. Comparison of three statistical methods used to analyse between‐species correlations. [file FEC-31-387-s006.pdf]

Table S3 Variance-covariance matrices for equation 1 for (a) all species together, (b) cereals only, (c) pulses only and for equation 2 for (d) all species together, (e) cereals only and (f) pulses only.

(a) Equation 1 – all species.

	**M0**	**lambda**	**d**	**Ar**	**c**
**M0**	1.00				
**lambda**	-0.76	1.00			
**d**	-0.08	-0.27	1.00		
**Ar**	0.53	-0.61	-0.27	1.00	
**c**	-0.36	0.25	0.18	-0.16	1.00

(b) Equation 1 – cereals only.

	**M0**	**lambda**	**d**	**Ar**	**c**
**M0**	1.00				
**lambda**	-0.34	1.00			
**d**	0.00	-0.86	1.00		
**Ar**	0.28	-0.11	-0.22	1.00	
**c**	-0.58	-0.03	0.13	0.08	1.00

(c) Equation 1 – pulses only.

	**M0**	**lambda**	**d**	**Ar**	**c**
**M0**	1.00				
**lambda**	-0.89	1.00			
**d**	0.31	-0.16	1.00		
**Ar**	0.42	-0.33	0.06	1.00	
**c**	-0.46	0.27	0.17	-0.17	1.00

(d) Equation 2 – all species.

	New seed mass	Total seed number
**New seed mass**	1.00	
**Total seed number**	-0.70	1.00

(e) Equation 2 – cereals only.

	Individual seed mass	Seed number per infructescence (Ns)	Infructescence number per plant (Ni)
**Individual seed mass**	1.00	-	
**Seed number per infructescence (Ns)**	-0.17	1.00	
**Infructescence number per plant (Ni)**	0.01	-0.58	1.00

(f) Equation 2 – legumes only.

	M0	Total seed number
**M0**	1.00	
**Total seed number**	-0.81	1.00

Table S4 Comparison of three statistical methods (pgls, lmekin and lme) used to analyse differences between crops and their progenitors. All traits were natural log transformed except *d*, *Ar*, *c* and $\tilde{\lambda}$.

Trait	pgls	lmekin	lme
Y – total seed yield	1.5 × higher *	1.7 × higher **	1.8 × higher ***
M_S – individual seed mass	1.9 × larger ***	1.8 × larger ***	1.9 × larger ***
d - duration of growth	NS	NS	NS
Ar - % reproductive mass	NS	NS	NS
c - proportion of chaff	37% less chaff **	38% less chaff ***	40% less chaff ***
Total aboveground biomass	1.4 × larger *	1.4 × larger **	1.5 × larger ***
$\tilde{\lambda}$ – relative growth rate	NS	NS	4% lower **
Total seeds per plant	NS	NS	NS
Seed number per gram plant biomass	NS	NS	1.4 x higher ***
Height	NS	NS	1.1 × taller**
**Cereals only**			
Infructescence mass	2.1 × larger ***	1.8 × larger ***	1.8 × larger ***
Ns - number seeds per infructescence	1.3 × more **	1.3 × more ***	1.3 × more ***
Ni - number of infructescences	NS	NS	NS

Table S5 Comparison of three statistical methods (pgls, lmekin and lme) used to analyse between-species correlations.

Correlation	pgls	lmekin	lme
ln (total seed yield) ~ ln (aboveground biomass)	$P < 0.0001$<br>Intercept = -1.1<br>Slope = 1.11<br>$R^2 = 0.86$	$P < 0.0001$<br>Intercept = -0.6<br>Slope = 0.85	$P < 0.0001$<br>Intercept = -1.1<br>Slope = 1.06
ln (total seed yield) ~ ln (individual seed mass)	$P < 0.001$<br>Intercept = -0.8<br>Slope = 0.58<br>$R^2 = 0.54$	$P < 0.0001$<br>Intercept = -0.1<br>Slope = 0.33	$P < 0.0001$<br>Intercept = -0.4<br>Slope = 0.59
ln (aboveground biomass) ~ ln (individual seed mass)	$P < 0.001$<br>Intercept = 0.3<br>Slope = 0.52<br>$R^2 = 0.60$	$P < 0.0001$<br>Intercept = 1.3<br>Slope = 0.21	$P < 0.0001$<br>Intercept = 1.4<br>Slope = 0.34
