# Supplementary material for: How did the domestication of Fertile Crescent grain crops increase their yields?
Source: Funct Ecol. 2016 Oct 3;31(2):387–97. doi: 10.1111/1365-2435.12760 (PMC5324541; doi:10.1111/1365-2435.12760)
Supplement: Supplementary file 7 — Table S6. Species means of traits measured in the yield experiments. [file FEC-31-387-s007.pdf]

Table S6 Species means (and standard error) for the traits measured in the yield experiments. Traits are sown individual seed mass (mg), total seed yield (g), total aboveground (vegetative + reproductive) biomass (g), % chaff, % reproductive biomass, duration of growth (days to flowering), height (cm), harvested individual seed mass (mg), total number of seeds per plant, relative growth rate ($g.g^{-1}.day^{-1}$), and for cereals only, infructescence mass (mg), number of infructescences per plant and the number of seeds per infructescence. Note that data for sown individual seed mass, total seed yield and total aboveground biomass are calculated using data from 2011 and 2013, whereas all other traits are calculated only from the 2011 experiment.

Species	Sown individual seed mass (mg)	Total seed yield (g)	Total aboveground biomass (g)	Chaff (%)	Reproductive aboveground biomass (%)	Duration of growth (days to flowering)	Height (cm)
Avena sativa	25.13 (1.29)	6.18 (0.63)	13.92 (1.04)	25.32 (1.28)	54.1 (1.6)	90.6 (2.2)	83.9 (3.6)
Avena sterilis	14.74 (1.62)	4.94 (0.98)	10.58 (1.15)	48.14 (1.95)	53.2 (1.8)	91.9 (2.6)	75.9 (2.9)
Hordeum vulgare subsp. *spontaneum*	27.33 (1.32)	5.33 (0.51)	13.01 (1.20)	41.24 (2.37)	59.1 (1.8)	102.9 (1.9)	51.7 (2.4)
Hordeum vulgare subsp. *vulgare*	39.72 (2.70)	9.48 (0.78)	15.99 (1.59)	12.85 (1.27)	65.2 (1.4)	99.1 (2.4)	52.4 (2.9)
Secale cereale subsp. *cereale*	28.06 (1.23)	5.79 (0.79)	15.79 (1.70)	22.31 (2.42)	41.8 (2.0)	90.9 (2.0)	98.3 (3.1)
Secale vavilovii	22.37 (1.36)	6.13 (0.91)	20.22 (1.53)	25.65 (3.95)	33.8 (2.5)	107.4 (1.3)	114.7 (5.4)
Triticum monococcum subsp. *aegilopoides*	16.68 (0.87)	2.03 (0.35)	8.02 (0.80)	40.70 (1.14)	48.8 (2.7)	109.2 (0.8)	63.5 (2.6)
Triticum monococcum	30.25 (1.11)	5.64 (0.42)	15.88 (0.79)	28.53 (1.62)	45.1 (1.8)	122.1 (1.3)	74.5 (2.5)

subsp. *monococcum*							
Triticum turgidum subsp. *dicoccoides*	26.07 (0.96)	3.72 (0.31)	13.55 (1.24)	51.85 (1.36)	54.6 (1.7)	107.4 (0.9)	60.0 (2.9)
Triticum turgidum subsp. *dicoccon*	35.88 (1.40)	5.64 (0.60)	18.15 (1.55)	28.29 (1.64)	47.5 (1.9)	102.3 (1.2)	75.9 (3.9)
Cicer arietinum	280.34 (19.21)	5.64 (0.46)	12.41 (0.69)	22.34 (5.15)	66.6 (1.3)	100.9 (0.9)	44.7 (1.6)
Cicer reticulatum	151.08 (7.24)	2.50 (0.21)	7.38 (0.43)	34.38 (1.90)	58.6 (2.2)	99.1 (0.6)	36.1 (1.3)
Lens culinaris subsp. *culinaris*	49.00 (3.11)	2.23 (0.29)	5.38 (0.53)	22.91 (4.83)	55.8 (2.9)	98.8 (0.9)	40.3 (1.6)
Lens culinaris subsp. *orientalis*	10.89 (0.90)	0.87 (0.12)	2.22 (0.23)	42.56 (5.91)	63.9 (3.4)	102.1 (0.0.7)	29.4 (17)
Pisum sativum subsp. *elatius*	72.52 (5.48)	6.86 (1.24)	10.05 (1.76)	21.73 (3.05)	62.6 (1.4)	97.5 (0.6)	95.3 (9.0)
Pisum sativum subsp. *elatius* var. *pumilio*	92.21 (5.20)	3.50 (0.55)	6.07 (0.68)	44.38 (9.36)	67.5 (1.2)	96.9 (0.4)	45.7 (3.7)
Pisum sativum subsp. *sativum*	173.92 (14.92)	5.72 (0.95)	11.46 (1.54)	30.52 (7.32)	67.8 (1.4)	102.2 (1.7)	74.9 (9.8)

Species	Harvested individual seed mass (mg)	Total seeds per plant	$\tilde{\lambda} -$ relative growth rate	Infructescence mass (mg)	Infructescence number	Seeds per infructescence
Avena sativa	27.94 (1.67)	246.5 (33.3)	0.0700 (0.0017)	1037.3 (93.3)	6.1 (0.4)	41.2 (5.1)
Avena sterilis	16.75 (1.95)	288.7 (48.5)	0.0730 (0.0029)	425.7 (59.6)	10.5 (1.1)	25.5 (1.8)
Hordeum vulgare subsp. *spontaneum*	25.19 (1.88)	204.4 (17.3)	0.0601 (0.0013)	330.8 (36.0)	16.0 (0.9)	12.6 (0.7)

Hordeum vulgare subsp. *vulgare*	41.13 (1.70)	196.5 (17.1)	0.0600 (0.0015)	715.2 (68.6)	11.9 (0.7)	18.2 (2.0)
Secale cereale subsp. *cereale*	32.45 (1.74)	169.0 (17.4)	0.0689 (0.0024)	646.8 (63.9)	10.1 (1.3)	20.9 (2.6)
Secale vavilovii	28.83 (1.91)	199.2 (23.0)	0.0640 (0.0009)	480.8 (59.7)	12.4 (0.8)	16.1 (1.6)
Triticum monococcum subsp. *aegilopoides*	12.80 (0.87)	154.9 (16.4)	0.0541 (0.0009)	260.7 (30.6)	7.1 (0.6)	22.5 (2.1)
Triticum monococcum subsp. *monococcum*	18.23 (0.84)	341.1 (19.1)	0.0514 (0.0006)	407.1 (22.6)	15.4 (0.8)	22.4 (0.8)
Triticum turgidum subsp. *dicoccoides*	30.37 (1.08)	119.6 (11.6)	0.0567 (0.0010)	479.7 (19.2)	7.6 (0.6)	15.9 (0.9)
Triticum turgidum subsp. *dicoccon*	28.89 (1.65)	219.3 (16.4)	0.0601 (0.0011)	686.2 (68.2)	9.5 (0.5)	23.0 (1.2)
Cicer arietinum	286.05 (31.49)	20.5 (1.9)	0.0382 (0.0010)	NA	NA	NA
Cicer reticulatum	172.17 (6.37)	16.9 (1.6)	0.0396 (0.0008)	NA	NA	NA
Lens culinaris subsp. *culinaris*	57.49 (5.24)	46.2 (10.5)	0.0467 (0.0015)	NA	NA	NA
Lens culinaris subsp. *orientalis*	10.07 (0.57)	82.2 (14.0)	0.0542 (0.0014)	NA	NA	NA
Pisum sativum subsp. *elatius*	97.03 (8.21)	68.8 (11.1)	0.0489 (0.0013)	NA	NA	NA
Pisum sativum subsp. *elatius* var. *pumilio*	114.18 (4.86)	32.2 (5.7)	0.0416 (0.0015)	NA	NA	NA
Pisum sativum subsp. *sativum*	217.69 (6.95)	25.8 (5.9)	0.0349 (0.0016)	NA	NA	NA
